# Tuning the Hsp70 chaperone cycle: emerging roles of GrpE-like nucleotide exchange factors in proteostasis and organelle function

**DOI:** 10.1093/procel/pwaf086

**Published:** 2025-10-24

**Authors:** Marc A Morizono, Tiffany V Safar, Mark A Herzik

**Affiliations:** Department of Chemistry and Biochemistry, University of California, San Diego, La Jolla, CA 92093, United States; Department of Chemistry and Biochemistry, University of California, San Diego, La Jolla, CA 92093, United States; Department of Chemistry and Biochemistry, University of California, San Diego, La Jolla, CA 92093, United States

**Keywords:** heat shock protein 70, GrpE, nucleotide exchange factor, proteostasis, chaperones

## Abstract

The heat shock protein 70 (Hsp70) family of molecular chaperones is essential for nearly every cell to support protein homeostasis through folding, signaling, and quality control. Hsp70 functionality critically depends on co-chaperones, including the GrpE-like family of nucleotide exchange factors (NEFs), first identified in *Escherichia coli* as GrpE. These factors have long been recognized for their ability to catalyze the release of Hsp70 nucleotide and protein substrates, but recent structural and functional studies have revealed that GrpE-like NEFs are more than passive exchange catalysts, instead acting as dynamic regulators that coordinate chaperone activity with cellular stress responses, organelle-specific demands, and allosteric control of substrate binding and release. In this review, we synthesize decades of research on GrpE-like proteins across bacteria and eukaryotes, culminating in high-resolution structures of the human mitochondrial NEF, GrpEL1, in complex with mitochondrial Hsp70. We examine how architectural features of GrpE-like NEFs have evolved to meet specialized demands, such as thermosensing in bacteria, redox-responsive regulation in vertebrates, and coordination of protein import in mitochondria. We further describe how discrete structural domains dynamically control chaperone cycling, including nucleotide and substrate release, and how gene duplication and domain specialization have driven functional diversification in higher eukaryotes. Finally, we highlight emerging evidence linking NEF activity to mitochondrial homeostasis, stress adaptation, and disease, reframing GrpE-like NEFs as tunable regulators rather than static cofactors. This perspective positions them as stress-adaptive control points in proteostasis and offers a conceptual framework for understanding how ancient chaperone systems have evolved to meet the regulatory needs of modern and complex eukaryotic cells.

## Introduction

**Figure 1.**
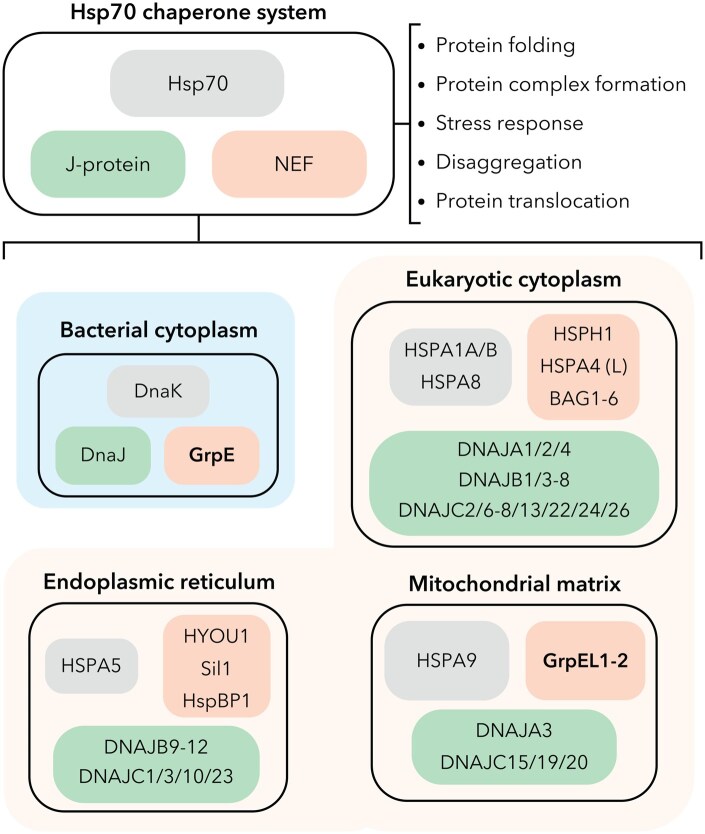
**Conservation and diversity of Hsp70 chaperone systems in biology**. (Top) The Hsp70 chaperone system comprises a core Hsp70 protein that collaborates with J-domain proteins and nucleotide exchange factors (NEF), enabling proteostasis. (Bottom) Gene names of Hsp70 proteins (e.g., DnaK, HSPA1/B, HSPA8, ER-resident HSPA5, and mitochondrial HSPA9/mtHsp70) are shown in gray, J-domain proteins (e.g., DNAJ/HSP40 family, and mitochondrial DNAJC19/Pam18) are shown in green, and the four NEF families (GrpE-like, Hsp110/Grp170, HspBP1/Sil1, and BAG1–6) are shown in coral with members of the GrpE-like NEF family bolded. Bacterial systems (represented by *Escherichia coli*) are boxed in blue, and eukaryotic systems (represented by *Homo sapiens*) are boxed in beige.

The heat shock protein 70 (Hsp70) chaperone system represents a ubiquitous means for cells to maintain protein homeostasis, otherwise referred to as proteostasis, with essential roles in protein folding, stress responses, translocation, degradation, and quality control. The ubiquity of the Hsp70 chaperone system in nearly all known organisms highlights its essential role in supporting cellular homeostasis across all kingdoms of life (Calloni et al., 2012; Fernández-Fernández et al., 2017; Macario et al., 2006). In bacteria, a single Hsp70 system, termed DnaK, suffices for general proteostasis, while eukaryotes have evolved compartment-specific Hsp70 isoforms (Calloni et al., 2012; Schönfeld et al., 1995). These include endoplasmic reticulum (ER)-resident binding immunoglobulin protein (HSPA5/BiP/Grp78), cytoplasmic Hsp70 (HSPA1A/B) and heat shock cognate 70 (HSPA8/Hsc70) paralogs, and mitochondrial Hsp70 (HSPA9/mtHsp70/Grp75/mortalin), each with tailored J-domain protein(s) (J-protein/Hsp40s) and nucleotide exchange factor (NEF) co-chaperones that facilitate adenosine triphosphate (ATP) hydrolysis and nucleotide/substrate exchange, respectively (Fig. 1) (Bracher and Verghese, 2023; Kampinga and Craig, 2010; Rosenzweig et al., 2019). Despite conserved ATPase-driven mechanisms, these systems exhibit striking regulatory diversity and substrate specificity, reflecting their adaptation to distinct proteostatic demands within different cellular environments. In this review, we refer to representative organisms when summarizing findings—e.g., bacteria (*Escherichia coli*), yeast (*Saccharomyces cerevisiae*), and human (*Homo sapiens*).

**Figure 2.**
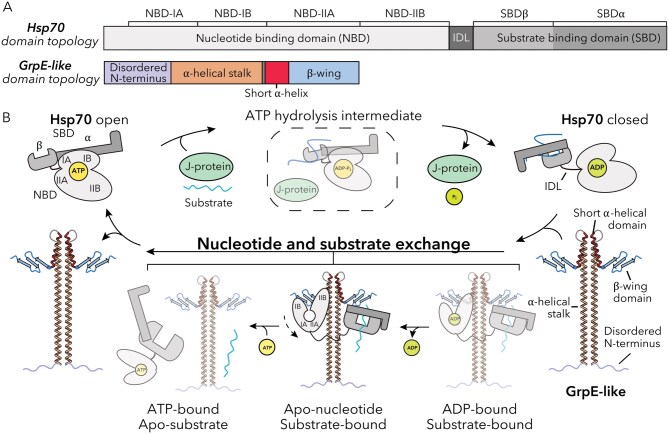
**GrpE-like co-chaperone regulation of the Hsp70 chaperone catalytic cycle**. (A) Domain topologies of Hsp70 (top) and GrpE-like (bottom) colored by key regions. (B) Catalytic cycle of Hsp70 mediated by GrpE-like NEFs with key steps indicated. Intermediate states that are speculated to exist but have not been experimentally shown are indicated by semi-transparency. A J-domain protein representation is depicted (green) to illustrate stimulation of Hsp70 ATPase activity.

All known Hsp70s characterized to date harbor the same topology: a N-terminal nucleotide-binding domain (NBD) that is responsible for ATP binding and hydrolysis, with a C-terminal protein substrate-binding domain (SBD) separated by a short interdomain linker (IDL) (Fig. 2A) (Fernández-Fernández et al., 2017; Kampinga and Craig, 2010; Rosenzweig et al., 2019). In its canonical cycle, Hsp70 transitions between nucleotide-dependent open and protein/peptide substrate-bound closed states. In the open state, Hsp70 adopts a compact conformation in which an ATP-bound NBD and apo SBD are tightly associated, with the substrate-binding pocket of the SBD open and exposed to solvent (Fig. 2B). Upon peptide substrate binding to the SBD, ATP hydrolysis in the NBD is stimulated by a J-protein co-chaperone, triggering large allosteric changes that close the SBD to capture the protein substrate. In the closed state, the NBD and substrate-bound SBD are physically separated but remain tethered by the flexible IDL. Here, multivalent interactions with a NEF, GrpE-like in bacteria, mitochondria, and chloroplasts, facilitate adenosine diphosphate (ADP) release via mechanical opening of the NBD and allow for subsequent binding of ATP (Kityk et al., 2018; Szabo et al., 1994). The Hsp70-GrpE complex has been shown to be ATP-sensitive, with the addition of ATP resulting in rapid substrate release and dissociation of Hsp70 from GrpE. Importantly, ATP binding at the NBD is thought to initiate restructuring of Hsp70 back to the open state, resetting the chaperone for a new cycle of substrate binding (Fig. 2B) (Mayer and Bukau, 2005; Voos et al., 1994). Although this co-chaperoning function of GrpE-like proteins is conserved, these mechanisms have been assimilated into additional functions to meet diverse environmental needs (Craig, 2018; Grimshaw et al., 2001; Konovalova et al., 2018; Schmidt et al., 2010). For completeness, a J-domain protein representation is shown schematically in Fig. 2B to emphasize its role in ATPase stimulation, although the function of J-domain proteins will not be the focus of this review.

Investigations into the mechanism of action of GrpE-like NEFs have spanned more than three decades since the first NEF from *E*. *coli*, growth requirement protein E (GrpE), was identified in the late 1980s (Zylicz et al., 1987, 1989). Early studies elucidated the mechanism of nucleotide exchange in *E*. *coli* Hsp70 (DnaK) and other bacterial species, yet the role of GrpE proteins in facilitating substrate release has remained incomplete (Harrison et al., 1997). Furthermore, structural insights have been restricted to bacterial systems due to challenges in determining eukaryotic GrpE and Hsp70 structures (Harrison et al., 1997; Nakamura et al., 2010; Wu et al., 2012; Xiao et al., 2024). However, recent structural elucidation of human mitochondrial Hsp70 (mtHsp70/mortalin) in complex with the mitochondrial-specific NEF, GrpEL1, has provided a near-complete mechanism of GrpE(L1) function, allowing for in-depth comparative analyses of GrpE-like proteins across kingdoms (Morizono et al., 2024). In this review, we will examine the structural and functional diversification of GrpE-like NEFs, highlighting how these differences have evolved to support increasingly specialized cellular processes, particularly in higher eukaryotes. We will also discuss the remaining knowledge gaps of GrpE-like function related to regulation in response to cellular stress and roles in mitochondrial import processes.

## Evolutionary origins and diversification of GrpE-like NEFs

Generally, NEFs facilitate the exchange of nucleoside diphosphates for nucleoside triphosphates — a process essential for numerous cellular functions requiring nucleotide exchange (Mayer and Bukau, 2005). NEFs comprise four structurally diverse families that have emerged to augment Hsp70 regulation, including GrpE-like, Hsp110/Grp170, HspBP1/Sil1, and BAG domains (Fig. 1) (Alberti et al., 2003; Bracher and Verghese, 2023; Dragovic et al., 2006; Harrison, 2003; Shomura et al., 2005). While the localization and functional roles beyond nucleotide exchange differ for each NEF family, each of these families employs a distinct structural strategy to remodel the NBD of their Hsp70 partners to enable nucleotide release. Hsp110/Grp170 proteins are large Hsp70 relatives that act as both holdases and NEFs, stabilizing unfolded substrates while promoting ADP release through a conserved NBD–NBD interface (Dragovic et al., 2006). HspBP1 and its ER paralog Sil1 use armadillo-repeat folds to pry open the NBD (Shomura et al., 2005). BAG proteins, in contrast, employ a short α-helical BAG domain to bind and stabilize an open NBD conformation, often coupled to signaling modules that link Hsp70 activity to apoptosis, growth factor signaling, or proteasomal degradation (Alberti et al., 2003). Compared to these families, GrpE-like NEFs are unique in their dimeric architecture and in their capacity to regulate both nucleotide and protein substrate release simultaneously. This distinction has enabled members of the GrpE-like family, found in bacteria, mitochondria, and chloroplasts, to adopt additional roles in protein translocation and oxidative stress responses with specific eukaryotic isoforms (Bracher and Verghese, 2023; Craig, 2018; Harrison, 2003; Konovalova et al., 2018).

The founding member of the GrpE-like family, GrpE from *E*. *coli*, plays a central role in regulating the activity of bacterial DnaK/Hsp70 and is structurally and functionally conserved across prokaryotes (Harrison, 2003). As mitochondria evolved from an alphaproteobacterial endosymbiont, many of the genes encoding chaperones and their cofactors were adapted to accommodate organellar integration via gene duplication, gene loss, and lateral gene transfer (Craig, 2018; Hewitt et al., 2011, 2014; Roger et al., 2017). In most lineages, ancestral bacterial chaperones, including GrpE/DnaK, were lost from the organelle genome and replaced by nuclear-encoded homologs that acquired N-terminal mitochondrial targeting sequences (MTSs) for post-translational import into mitochondria. Conversely, direct import of extant bacterial GrpE into mitochondria has not been observed; instead, eukaryotic GrpE-like proteins (e.g., Mge1 in yeast and GrpEL1/2 in humans) fulfill the NEF role within the matrix. These remodeling events—gene loss/duplication, retargeting, and domain tuning—underpin the present compartment-specialized proteostasis systems (Allu et al., 2015; Jain et al., 2025; Roger et al., 2017).

These events ultimately engendered variants of bacterial machineries with functional adaptations characteristic of present-day mitochondria. This evolutionary adaptation gave rise to mitochondrial-specific GrpEL1, a eukaryotic NEF that retains the core structural features of bacterial GrpE but has acquired new functionalities to meet the demands of mitochondrial protein import and stress regulation via mitochondrial Hsp70 (Craig, 2018; Ma et al., 2022; Morizono et al., 2024). GrpEL1 is conserved across nearly all opisthokonts, including yeast and metazoans, and is considered the primary mitochondrial NEF in these organisms (Morizono et al., 2024; Neupane et al., 2022). In contrast, vertebrates possess a second mitochondrial GrpE paralog, GrpEL2, that likely emerged from a gene duplication event early in vertebrate evolution. Phylogenetic analyses reveal that while GrpEL1 orthologs are broadly conserved, GrpEL2 sequences are only detectable in vertebrate lineages (Konovalova et al., 2018; Srivastava et al., 2017).

The divergence of GrpEL2 from GrpEL1 enabled the acquisition of specialized features, including a potential redox-sensing function and unique tissue expression patterns, discussed below in greater detail (Konovalova et al., 2018; Srivastava et al., 2017). This functionalization event mirrors broader trends in the evolution of the mitochondrial proteostasis network, wherein core elements of the chaperone system have been repurposed to address compartment-specific roles. As such, the GrpE-like NEFs provide a model for studying how structural constraints and cellular demands shape the evolution of molecular machines. Ongoing efforts to map the distribution, sequence divergence, and functional specialization of GrpEL2 across vertebrates will shed light on the adaptive significance of this paralog and its emerging noncanonical roles.

## Mechanisms of GrpE-mediated nucleotide and substrate release from Hsp70s

**Figure 3.**
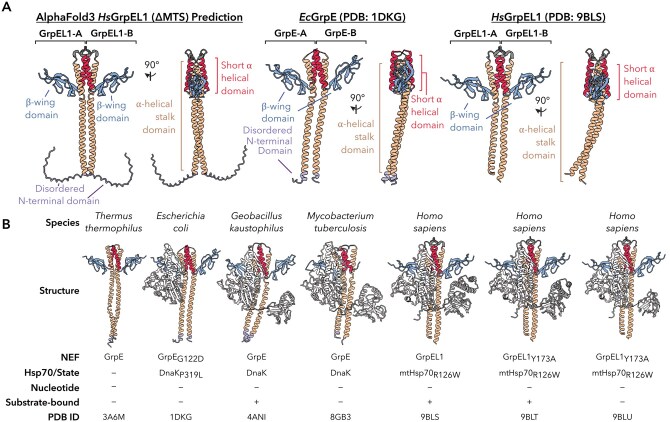
**Architecture of GrpE-like proteins and complexion with their Hsp70 partners**. (A) Annotated domains of GrpE-like proteins from predicted and experimentally determined structures. The AlphaFold3 *H. sapiens* GrpEL1 predicted (MTS removed, left) (Abramson *et al.*, 2024), *E. coli* GrpE experimental (PDB ID: 1DKG, middle) (Harrison *et al.*, 1997), and *H. sapiens* GrpEL1 experimental (PDB ID: 9BLS, right) (Morizono *et al.*, 2024) structures are colored according to Fig. 2A. (B) All currently available experimental structures of GrpE-like proteins. Domains are colored as in Fig. 2A.

GrpE-like proteins share a conserved architecture across kingdoms comprising a disordered N-terminus, an α-helical stalk domain forming a pseudo coiled-coil in the functional dimer, a short α-helical domain, and a β-rich C-terminal “winged” domain (Figs. 2A and 3A) (Morizono et al., 2024). Homodimerization through the α-helical stalk domain yields a cruciform-like architecture with the short α-helical domains acting as hinges to orient the β-rich winged domains outward (Fig. 3). While the GrpE-like topology is generally conserved, subtle structural variations across species and organellar isoforms confer unique functional properties. As most resolved structures of GrpE-like proteins are in complex with their Hsp70 partners, mechanistic insights into how the GrpE architecture engages the Hsp70 NBD and SBD to promote ADP and substrate release, respectively, can be gleaned (Fig. 3B).

Earlier structural studies of GrpE-like NEFs in bacteria (e.g., *E*. *coli*, *Thermus thermophilus*, *Geobacillus kaustophilus*, *Mycobacterium tuberculosis*) have provided foundational insights into the architecture and function of this family. Initial insights were obtained from the crystal structure of *E*. *coli* GrpE in complex with DnaK (Protein Data Bank [PDB] ID: 1DKG), revealing how the β-wing domain of GrpE engages the Hsp70 NBD to wedge apart its lobes, establishing the basic mechanistic framework for how GrpE-like proteins facilitate nucleotide release (Harrison et al., 1997). Subsequent structures of bacterial GrpE complexes, including those from *Thermus thermophilus* (PDB ID: 3A6M) (Nakamura et al., 2010), *Geobacillus kaustophilus* (PDB ID: 4ANI) (Wu et al., 2012), and *Mycobacterium tuberculosis* (PDB ID: 8GB3) (Xiao et al., 2024), highlighted the conserved cruciform dimeric architecture while capturing distinct conformations of the Hsp70 NBD and SBD. Together, these studies established that GrpE-mediated allostery extends across both the NBD and SBD. It is important to note, however, that many of the earlier structures relied on truncations of the GrpE N-terminal disordered domain, stabilizing mutations, or were unable to visualize full-length components due to proteolysis or dynamics, limiting the extent to which they captured dynamic states of the exchange cycle.

**Figure 4.**
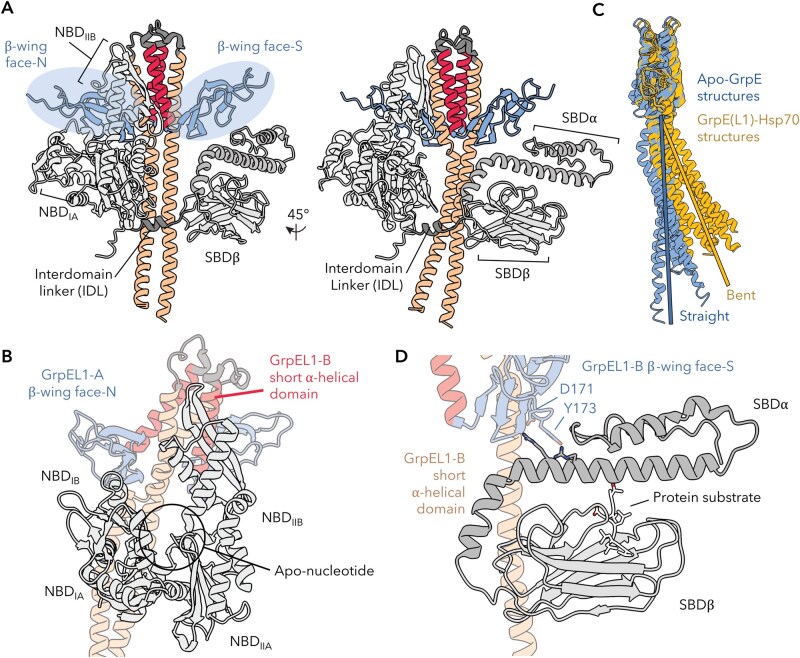
**GrpE-like proteins interact asymmetrically with Hsp70s to facilitate nucleotide release and substrate stabilization**. (A) Side and 45˚ rotated views of the human GrpEL1–mtHsp70 complex (PDB ID: 9BLS) showing asymmetric engagement of the β-wing-N and -S faces with the Hsp70 nucleotide-binding domain (NBD) and substrate-binding domain (SBD), respectively. (B) Top view highlighting the β-wing face-N of GrpEL1-A contacting the NBD IIA lobe and the short α-helical domain of GrpEL1-B stabilizing the IIB lobe in the apo-nucleotide state. PDB ID: 9BLS. (C) Comparison of apo-GrpE(L1) (blue; PDB IDs: 3A6M and 1DKG, and AlphaFold3 *Hs*GrpEL1 prediction) and mtHsp70-bound GrpEL1 (yellow; PDB IDs: 4ANI, 8GB3, 9BLS, 9BLT, 9BLU) structures reveals bending of the α-helical stalk upon Hsp70 binding. (D) Close-up of β-wing face-S from GrpEL1-B showing Asp171 and Tyr173 interacting with positively charged residues in the mtHsp70 SBDα, stabilizing substrate engagement.

More recently, full-length cryogenic electron microscopy (cryoEM) structures of the human GrpEL1-mtHsp70 complex in distinct states (PDB IDs: 9BLS, 9BLT, 9BLU) have expanded these bacterial observations into the mitochondrial system, providing the most complete mechanistic description of how GrpE-like proteins engage their Hsp70 partners to facilitate nucleotide and protein substrate exchange via asymmetric β-wing interfaces (Morizono et al., 2024). Consistent with prior studies, two GrpEL1 protomers interact with a single mtHsp70 molecule via an extensive interface spanning all Hsp70 domains: the NBD lobes IA/B and IIA/B, the IDL, and both β- and α-subdomains of the SBD (Figs. 2, 3B, and 4A). Despite the apparent symmetry of the GrpE-like dimer, the GrpEL1 protomers in GrpEL1–mtHsp70 complexes expose unique faces of the β-wing domain to mtHsp70, rendering the homodimeric GrpEL1 an asymmetric complex. This asymmetric architecture enables precise interactions with the multi-domain mtHsp70 molecule, whereby the mtHsp70 NBD interacts with one protomer of GrpEL1, designated GrpEL1-A, via the NBD-interacting face of the β-wing domain, termed β-wing face-N, and the SBD interacts with the other GrpEL1 protomer, designated GrpEL1-B, via the SBD-interacting face of the β-wing domain, termed β-wing face-S (Fig. 4A). Here, the NBD lobes IB and IIB are wedged apart via interactions with β-wing face-N and with the short α-helical domain of GrpEL1-B. Electrostatic and van der Waals interactions at these interfaces anchor GrpEL1 to the NBD lobes, stabilizing the apo-nucleotide NBD conformation (Fig. 4B). Downstream of the NBD, the conserved Hsp70 IDL interacts with the α-helical stalk domain, resulting in a distinct bend in the stalk (Fig. 4C). This bending enables simultaneous interactions between the β-wing face-S of GrpEL1-B and SBDα, stabilizing Hsp70 substrate binding. A conserved aspartic acid (Asp171 in human GrpEL1) and partially conserved tyrosine residue (Tyr173 in human GrpEL1) at this interface interact with positively charged arginine and lysine residues that extend from the SBDα subdomain (Fig. 4D). Thus, GrpEL1 coordinates nucleotide and substrate release by unlocking the NBD while stabilizing SBD substrate engagement, synchronizing both steps of the Hsp70 exchange cycle. ATP binding then triggers allosteric rearrangements that facilitate complex dissociation, substrate release, and resetting of Hsp70 for another round of chaperone activity (Moro et al., 2007; Xiao et al., 2024). Together, these complementary structures across bacteria and eukaryotic systems serendipitously sample distinct steps of the Hsp70 cycle, offering a patchwork of mechanistic insights that set the stage for full-length structural analyses. With the structural basis for GrpE-mediated nucleotide and substrate release fully visualized, these findings establish a mechanistic framework in which GrpE-like NEFs act as both catalytic and regulatory elements in the Hsp70 cycle, potentially tunable by redox state or cofactor remodeling.

## Functionally divergent roles of the disordered N-terminus

The N-terminus of GrpE proteins typically comprises 40–50 unstructured amino acids of varying length, complexity, and disparate functions (Figs. 2A and 3A) (Brehmer et al., 2004; Craig, 2018; Moro et al., 2007; Xiao et al., 2024). Previous studies have demonstrated that the N-terminus of bacterial GrpE contributes to substrate release from DnaK. Specifically, early studies observed that truncation of the *E*. *coli* GrpE N-terminus reduced its ability to stimulate substrate dissociation from DnaK (Brehmer et al., 2004; Harrison, 2003; Moro et al., 2007; Xiao et al., 2024). Therein, it was proposed that the disordered N-terminus may act as a pseudo-substrate for the substrate-binding site of Hsp70, thereby competing with and displacing bound substrate peptides. Although Hsp70s preferentially bind short, commonly leucine-rich hydrophobic peptides, the high local concentration of the negatively charged GrpE N-terminus may nonetheless assist with substrate displacement (Brehmer et al., 2004; Clerico et al., 2015; Mayer and Gierasch, 2019). However, to date, there is no direct structural evidence supporting physical insertion of the GrpE N-terminus into the SBD substrate-binding cleft. All high-resolution structures of bacterial GrpE–DnaK complexes show the GrpE N-terminus as disordered and unresolved, and the modeled interface between GrpE and DnaK places the N-terminus distal from the SBD (Fig. 3) (Harrison et al., 1997; Nakamura et al., 2010; Wu et al., 2012; Xiao et al., 2024). Thus, if substrate displacement occurs via the N-terminus, it likely requires a significant degree of local flexibility or partial disengagement from the β-wing domain, which normally orients the N-terminal stalks away from the SBD. Alternatively, the effect may be indirect, mediated by global conformational changes upon GrpE binding that allosterically destabilize substrate occupancy in the SBD.

Given the high sequence diversity of the GrpE-like N-terminus (31.6% identical across *E*. *coli*, *T. thermophilus*, *G*. *kaustophilus*, *M*. *tuberculosis*, *S*. *cerevisiae*, *H*. *sapiens*, *R*. *norvegicus*, *M*. *musculus*, *P. abelii*, and *B. taurus*), it remains unclear whether all GrpE homologs have this capability. In eukaryotic homologs, such as mitochondrial GrpEL1 and GrpEL2, this mechanism remains speculative. In particular, in mitochondrially targeted GrpE species, the first 30 amino acids of the ∼50 amino acid disordered N-terminal domain encode for an MTS that directs the protein through the import pathway to the mitochondrial matrix (MM) (Morizono et al., 2024; Schmidt et al., 2010; Srivastava et al., 2017). Following import through the inner mitochondrial membrane (IMM), mitochondrial processing peptidase (MPP) cleaves the MTS and allows for entry into the MM (Schmidt et al., 2010). The remaining N-terminal residues are highly charged and do not appear to be conserved in bacterial species. To date, structural evidence of the disordered N-terminus interacting with the SBD, in either bacterial or eukaryotic Hsp70-GrpE structures, is lacking and thus precludes definitive conclusions about its role in substrate displacement.

Taken together, while the bacterial GrpE N-terminus may act as a pseudo-substrate under certain conditions, such a mechanism has not been directly visualized and appears structurally constrained. In mitochondrial GrpEL(1/2) proteins, the absence of an extended, conserved, and charged N-terminal region, combined with recent structural data, refutes a conserved substrate displacement function. Nevertheless, further functional studies employing N-terminally modified GrpEL1/2 variants would be valuable to investigate the possibility of its potential role in eukaryotic Hsp70 substrate displacement.

## The elongated pseudo-coiled-coil α-helical domain’s role in dimerization and Hsp70 binding

The elongated α-helical stalk domain characteristic of GrpE proteins spans approximately 70 amino acids (∼20 α-helical turns) and adopts a pseudo coiled-coil domain in the functional dimer, contributing extensively to the stabilization of the homodimer through extensive buried surface area (>2,000 Å^2^) (Figs. 2–4). This domain is highly conserved across all GrpE members, with several residues showing high similarity or absolute conservation throughout the region (47.3% identical across *E*. *coli*, *T*. *thermophilus*, *G*. *kaustophilus*, *M*. *tuberculosis*, *S*. *cerevisiae*, *H*. *sapiens*, *R*. *norvegicus*, *M*. *musculus*, *P*. *abelii*, and *B*. *taurus*). In contrast to canonical coiled-coil domains, the GrpE-like pseudo coiled-coil is composed of two parallel alpha helices that are stabilized via internally facing hydrophobic residues and do not exhibit the ultra-twist characteristic of coiled-coil domains (Fig. 3) (Harrison et al., 1997; Morizono et al., 2024; Nakamura et al., 2010; Wu et al., 2012; Xiao et al., 2024). Interestingly, the degree of bending of the helices varies among available experimental structures and is significantly influenced by interactions with an Hsp70 binding partner. In the crystal structure of *T*. *thermophilus* GrpE (PDB ID: 3A6M) (Nakamura et al., 2010), the α-helical stalk appears nearly in-plane with the rest of the protein, exhibiting only minor kinking. Similarly, in the crystal structure of *E*. *coli* GrpE in complex with the NBD of DnaK (PDB ID: 1DKG) (Harrison et al., 1997), the long alpha helices remain straight and do not appear to extensively interact with the DnaK NBD. In contrast, structures of GrpE in complex with Hsp70s that contain the IDL and SBD regions (PDB IDs: 4ANI, 8GB3, 9BLS, 9BLT, and 9BLU) (Morizono et al., 2024; Wu et al., 2012; Xiao et al., 2024) show distinct bending (Fig. 4C). In these cases, regions of the GrpE α-helical stalk interact with the IDLs of Hsp70 proteins via hydrophobic interactions in bacteria and lower eukaryotes, or via salt bridges in higher eukaryotes. These interactions can induce significant bending, up to >25°, of the GrpE(L1) stalk relative to the plane of the GrpE(L1) protein (Morizono et al., 2024). Structural analysis of GrpE-Hsp70 complexes suggests that the magnitude of this bending may depend on interactions with the β-subdomain of the SBD, further accentuating the bend (Morizono et al., 2024; Xiao et al., 2024).

The short α-helical domain follows the α-helical stalk domain and is bridged by a short loop. This region is moderately conserved (32.2% identical across *E*. *coli*, *T*. *thermophilus*, *G*. *kaustophilus*, *M*. *tuberculosis*, *S*. *cerevisiae*, *H*. *sapiens*, *R*. *norvegicus*, *M*. *musculus*, *P*. *abelii*, and *B*. *taurus*) and consists of about six α-helical turns in both bacterial and eukaryotic species (Figs. 2A and 3A). Packing interactions between the paired short α-helices create a defined crossing angle that reinforces the cruciform-like architecture of the dimer, extending beyond the stalk domain. These interactions include conserved hydrophobic residues that interdigitate across the dimer interface, as well as salt bridges that further anchor the helices (Morizono et al., 2024). This stabilizing role is particularly important given the overall flexibility of GrpE, as it provides a potential pivot point that constrains the relative orientation of the β-wing domains away from the axis of dimerization. By fixing the spatial arrangement of the β-wings, the short α-helical domain ensures that each protomer presents a complementary surface to Hsp70, thereby enabling asymmetric engagement of the NBD and SBD. Interactions between the short alpha helices of each GrpE protomer contribute to the stabilization of the IIB NBD lobe in Hsp70. These stabilizing interactions include salt bridges, van der Waals interactions, and hydrophobic contacts, primarily bridging GrpE-B with the IIB NBD lobe (Fig. 4B). Along with interactions at the β-wing face-N, these contacts result in the opening of the Hsp70 NBD, exposing the nucleotide-binding pocket and facilitating ADP release. These interactions are observed in both bacterial and human GrpE-Hsp70 structures and represent the primary mechanism of ADP dissociation from the NBD.

## Dual faces of the asymmetric β-wing domains

The β-wing domains are the most C-terminal regions of GrpE and resemble wings protruding from the short α-helical domain, away from the dimer interface, resulting in a cruciform-like architecture. This domain is highly conserved (52.0% identical across *E*. *coli*, *T*. *thermophilus*, *G*. *kaustophilus*, *M. tuberculosis*, *S. cerevisiae*, *H. sapiens*, *R. norvegicus*, *M. musculus*, *P. abelii*, and *B. taurus*) and forms crucial contacts with the NBD and SBD of Hsp70, which are essential for Hsp70’s chaperoning activity (Figs. 2A, 3A, and 4A). Recent structural evidence has shown that each face of the human GrpEL1 β-wing domain, β-wing face-N and β-wing face-S, is distinct (PDB IDs: 9BLS, 9BLT, 9BLU) (Morizono et al., 2024). This pseudo-symmetry of the GrpEL1 dimer appears critical in ensuring proper orientation and stoichiometry of the mtHsp70 interaction. Here, interaction of the mtHsp70 NBD with β-wing face-N positions the IDL and SBD for further stabilizing interactions with the α-helical stalk and β-wing face-S, respectively (Fig. 4). Moreover, these interactions, inducing bending of the α-helical stalk, may prevent binding of an additional mtHsp70 unit on the opposing solvent-exposed GrpEL1 interface. Collectively, the pseudo-symmetric architecture of the GrpEL1 dimer and non-equivalent β-wing surfaces enable asymmetric engagement of a single Hsp70 protomer and ensure simultaneous access to both the NBD and SBD without steric clash.

Given the high sequence conservation of the β-wing domain across species and unique configuration of the exposed β-wing faces, it is likely that these solvent-exposed residues play critical roles in correctly orienting the distinct Hsp70 domains in homologous systems. While full-length structures of bacterial GrpE-Hsp70 complexes have yet to be resolved, existing experimental structures support the structural conservation of an asymmetric complex mediated by a pseudo-symmetric GrpE-like protein (Fig. 3).

## Bacterial GrpE can act as a thermosensor

**Figure 5.**
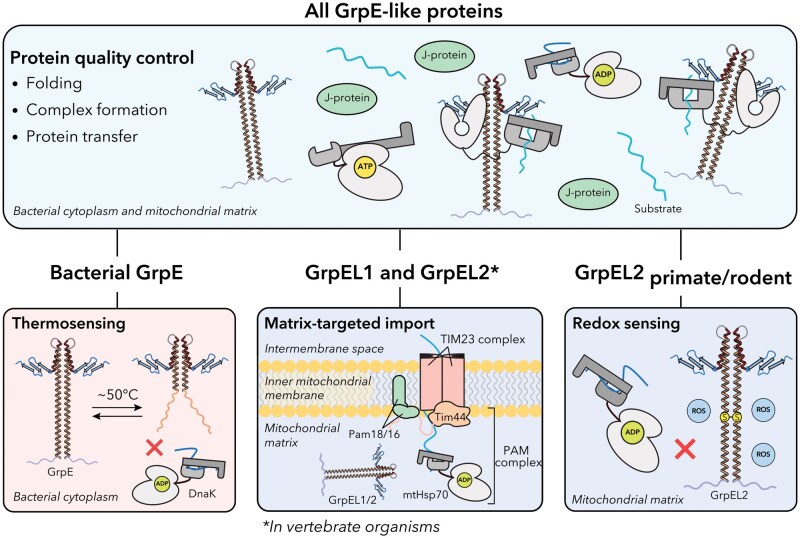
**Functional diversification of GrpE-like proteins**. (Top) All GrpE-like proteins act as nucleotide exchange factors for Hsp70 chaperones, promoting protein folding, complex assembly, and substrate transfer in the bacterial cytoplasm or mitochondrial matrix. (*Bottom left*) In bacteria, GrpE also functions as a thermosensor: reversible unfolding of the long α-helical stalk at elevated temperatures (∼50°C) inhibits ADP release from DnaK, allowing retention of aggregation-prone substrates under heat stress. (Bottom middle) In eukaryotes, GrpEL1, and GrpEL2 in vertebrates, are targeted to the mitochondrial matrix, where GrpEL1 is an essential component of the presequence translocase-associated motor (PAM) complex. Here, GrpEL1 recharges mtHsp70 during protein import in cooperation with Pam18, Pam16, and Tim44. (Bottom right) In vertebrates, GrpEL2 may function as a redox sensor: oxidative stress promotes disulfide bond formation between conserved cysteine residues, reducing interaction with ADP-bound mtHsp70 and potentially modulating substrate release in ROS-rich environments.

Beyond its role as a NEF for Hsp70, GrpE has also been described as a thermosensor where *E. coli* GrpE was shown to exhibit non-Arrhenius behavior, with decreased co-chaperone activity at elevated temperatures (Grimshaw et al., 2001). Specifically, two thermal transitions were observed: the first was described as a fully reversible conformational change with a midpoint at ∼50°C, and the second, a partially irreversible transition near 75°C (Fig. 5). These findings were later elaborated to determine that the first transition resulted from an unwinding of the α-helical stalk domain of GrpE, and the second transition resulted from unfolding of the shorter α-helical domain (Gelinas et al., 2002). Based on these results, the α-helical stalk was proposed to function as the thermosensing domain via a reversible and gradual helix-to-coil transition. A similar phenomenon—two thermal transitions (at 90°C and 105°C)—was also observed in *T. thermophilus* GrpE, a homolog from a heat-tolerant extremophile (Groemping and Reinstein, 2001). However, it was proposed that a different unfolding sequence occurred, with the β-wings unfolding first while the α-helical stalk enabled reversible refolding of the β-wings. While this discrepancy may reflect adaptations specific to thermophilic organisms, it nonetheless supports the idea that bacterial GrpEs possess thermosensing capabilities mediated by the reversible unfolding of specific structural domains. Structural information of bacterial Hsp70-GrpE complexes further supports this hypothesis, revealing interactions between GrpE’s α-helical stalk and the IDL of Hsp70 (Wu et al., 2012; Xiao et al., 2024). A helix-to-coil transition in this region would likely disrupt this interaction, impairing GrpE-mediated nucleotide exchange and substrate release. Under heat stress conditions, such inhibition could be beneficial, allowing Hsp70s to retain aggregation-prone substrates. Thus, reversible unfolding of GrpE may act as a regulatory mechanism that enables substrate sequestration during stress while maintaining GrpE levels required for basal activity. Although eukaryotic GrpE homologs may exhibit some sensitivity to thermal changes, their potential role as thermosensors remains largely unexplored. Instead, research has focused on their co-chaperone functions and emerging noncanonical roles (Fig. 5).

## GrpE-like NEFs have diversified to accommodate increasing environmental demands

Although the mechanism by which GrpE-like proteins interact with their Hsp70 binding partners to facilitate nucleotide and substrate exchange has been defined, GrpE-like species have evolved to adopt additional context-specific functions. Notably, GrpEL1 has acquired an additional essential role involving the import of proteins targeted to the MM via the presequence translocase-associated motor (PAM) complex (Fig. 5) (Michaelis et al., 2022; Neupane et al., 2022; Srivastava et al., 2017). This dual functionality highlights a specialized adaptation of GrpEL1 within the mitochondrial environment in addition to the regulation of mtHsp70 for protein quality control. Furthermore, vertebrates uniquely possess a second mitochondrial GrpE paralog termed GrpEL2 that, like GrpEL1, is thought to participate in the mtHsp70 chaperone system. Intriguingly, GrpEL2 has also been implicated in oxidative stress sensing, underscoring a potential functional adaptation specific to vertebrate species (Konovalova et al., 2018; Michaelis et al., 2022; Srivastava et al., 2017). However, the significance of GrpEL2’s co-chaperoning role relative to GrpEL1 remains incompletely defined, as evidence has shown GrpEL1, but not GrpEL2, to be indispensable for mitochondrial homeostasis.

## The PAM complex and GrpEL1

Within the MM, GrpEL1 not only functions as a canonical NEF for mtHsp70, but also serves as an essential component of the MM-localized PAM complex, which powers the import of nuclear-encoded preproteins bearing an MTS (Craig, 2018; Michaelis et al., 2022). The PAM complex operates downstream of the translocase of the IMM-23 (TIM23) complex and is responsible for the ATP-dependent translocation of matrix-destined polypeptides across the inner membrane (Fig. 5). In mammals, the core components of the PAM complex include HSPA9 (mtHsp70/Grp75/mortalin), GrpEL1, DNAJC19 (Pam18), Magmas (Pam16), and TIMM44 (Tim44) (Craig, 2018; Hewitt et al., 2014; Michaelis et al., 2022). In *S. cerevisiae*, these correspond to Ssc1 (mtHsp70), Mge1 (GrpE-like NEF), Pam18 (Tim14), Pam16 (Tim16), and Tim44 (Den Brave et al., 2024; Gill-Hille et al., 2022; Van Der Laan et al., 2005). Pam18 is a J-domain protein that stimulates the ATPase activity of mtHsp70, while Pam16 acts as a regulatory partner that modulates this activity (Craig, 2018; Hutu et al., 2008; Li et al., 2004; Truscott et al., 2003). Tim44, a peripheral membrane protein, serves as a scaffold that anchors the mtHsp70-GrpEL1 machinery to the IMM and coordinates the handoff of preproteins emerging from the translocase channel (Fig. 5) (Craig, 2018; Michaelis et al., 2022). During import, presequence-bearing preproteins exiting TIM23 are captured by mtHsp70 localized via Tim44. Subsequent ATP hydrolysis, stimulated by Pam18, enables capture of an imported preprotein, which is followed by nucleotide exchange mediated by GrpEL1 (Craig, 2018; Hewitt et al., 2014; Jain et al., 2025; Li et al., 2004; Morizono et al., 2024; Van Der Laan et al., 2005). Importantly, GrpEL1 plays the critical role of recharging mtHsp70 during preprotein import by promoting the release of ADP and facilitating ATP rebinding, thereby resetting the chaperone for successive rounds of substrate capture for iterative “pulling” cycles that facilitate translocation across the IMM (Morizono et al., 2024; Wu et al., 2012; Xiao et al., 2024).

Genetic depletion of GrpEL1 in model organisms results in profound defects in matrix protein import, leading to protein aggregation, mitochondrial dysfunction, and organismal lethality (Ma et al., 2022; Manjunath et al., 2024; Neupane et al., 2022; Srivastava et al., 2017). Beyond translocation, mtHsp70/GrpEL1 contributes indirectly to matrix protein folding and the biogenesis of multi-subunit assemblies, including respiratory chain complexes, by maintaining import competence and assisting maturation of select assembly factors, leading to defects during mtHsp70/GrpEL1 dysfunction/loss (Jain et al., 2025; Needs et al., 2021; Poveda-Huertes et al., 2021; Priesnitz et al., 2022). While the biochemical role of GrpEL1 in nucleotide exchange is well established, the precise nature of its integration into the PAM complex remains incompletely defined. Co-immunoprecipitation and crosslinking studies suggest that GrpEL1 physically associates with Tim44, raising the possibility that GrpEL1 may be spatially restricted within the matrix to enhance the efficiency of import cycles (Srivastava et al., 2017). Moreover, it remains unclear how GrpEL1 simultaneously interacts with mtHsp70 and other PAM components, such as Pam18 or Pam16, during protein translocation. The extent to which GrpEL1 functions in the PAM complex is modulated by redox status, import load, or cellular stress also remains an open question. Future work combining *in situ* structural analysis, proximity labeling, and time-resolved import assays will be essential for delineating how GrpEL1 is functionally and spatially integrated into the PAM machinery.

## Vertebrates harbor two GrpE-like homologs with specialized roles

**Figure 6.**
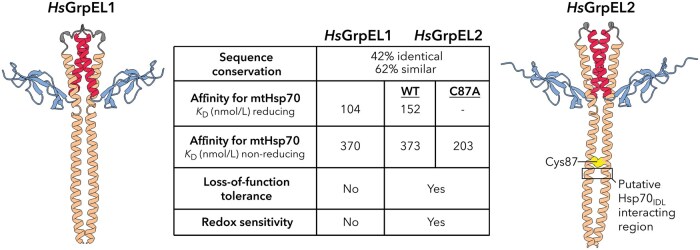
**Vertebrates harbor two distinct GrpEL isoforms**. Despite having high sequence and structural similarity, human GrpEL1 and GrpEL2 exhibit differences in affinity for mitochondrial Hsp70 (mtHsp70), essentiality, and potential redox sensitivity. A reported redox-sensitive cysteine (Cys87) in human GrpEL2 locates just upstream of the putative Hsp70_IDL_ interacting region (Konovalova *et al.*, 2018). Disulfide formation here may preclude mtHsp70 interaction.

The eukaryotic GrpE homologs, GrpEL1 and GrpEL2, are differentially found in eukaryotes, with GrpEL1 ubiquitous in higher eukaryotes (e.g., *S. cerevisiae* Mge1; *Drosophila* GrpE; mammalian GrpEL1), whereas concurrent GrpEL1 and GrpEL2 expression is specific to vertebrates (e.g., mouse, zebrafish, human) (Konovalova et al., 2018; Manjunath et al., 2024; Neupane et al., 2022; Srivastava et al., 2017). In humans, these paralogs share 42% sequence identity and 62% sequence similarity, and both follow the mitochondrial presequence import pathway, with final maturation occurring in the MM. Functionally, both GrpEL1 and GrpEL2 can act as NEFs in the canonical Hsp70 chaperone cycle, promoting protein homeostasis through direct interaction with mtHsp70 (Srivastava et al., 2017). A recent study examined the binding affinities of GrpEL1 and GrpEL2 for mtHsp70, and found that human GrpEL1 had approximately 10-fold higher affinity than GrpEL2 for ADP-bound mtHsp70, suggesting a preferred NEF in the MM (Manjunath et al., 2024). It was later demonstrated that GrpEL2 is dispensable in cultured human cells, whereas loss-of-function variants of GrpEL1 are not tolerated (Konovalova et al., 2018). In a mouse model, it was observed that a GrpEL1 knockout was embryonically lethal, and muscle-specific deletion resulted in rapid skeletal muscle atrophy and disorganization (Fig. 6) (Neupane et al., 2022). Despite their high sequence and structural similarity, GrpEL1 emerges as the essential and primary mitochondrial NEF. Nevertheless, the distinct properties of GrpEL2 suggest specialized roles that may uniquely contribute to vertebrate biology.

## Specific GrpEL2 species may play a role in oxidative stress sensing

A unique feature of GrpEL2 that has garnered recent interest is its potential role as an oxidative sensor. In a recent study, it was demonstrated that GrpEL2 oligomerization in HEK293 cells is sensitive to H_2_O_2_ treatment (Konovalova et al., 2018). They identified Cys87, located in the α-helical stalk domain, as the residue responsible for disulfide bond formation between GrpEL2 protomers. Notably, Cys87 is conserved only in primate and rodent GrpEL2. In a follow-up study, it was determined that under non-reducing conditions, mutation of Cys87 to an alanine residue increased its binding affinity to mtHsp70 almost 2-fold compared to wild-type, suggesting disulfide bond formation at Cys87 reduces GrpEL2’s affinity for ADP-bound mtHsp70 (Manjunath et al., 2024). Structurally, the formation of a disulfide bond at this position could rigidify the GrpEL2 pseudo coiled-coil, maintaining a straight conformation that restricts bending of the α-helical stalk and limits interactions with the IDL of mtHsp70 (Fig. 6). This hypothesis is supported by structural data from GrpE(L1)-Hsp70 complexes, which show that flexibility and bending of the GrpE(L1) α-helical stalk are critical for full complex formation (Morizono et al., 2024; Wu et al., 2012; Xiao et al., 2024). Similar to the thermosensing mechanism of bacterial GrpE, reduced interaction between substrate-bound Hsp70 and GrpEL2 under oxidative conditions could prevent premature release of aggregation-prone substrates, maintaining protein homeostasis. Given Cys87’s exclusivity to GrpEL2 in primates and rodents, it has been proposed that this oxidative regulation may have evolved in longer-lived species with higher metabolic activities and elevated levels of reactive oxygen species (ROS) (Manjunath et al., 2024). However, further studies are needed to determine whether physiological levels of oxidative stress are sufficient to induce disulfide bond formation and how such conditions affect the co-chaperone functions and abundance of both GrpEL1 and GrpEL2.

Moreover, a parallel, well-studied redox regulation that exists in the *S. cerevisiae* mitochondrial NEF Mge1 provides rationale for GrpEL2’s proposed thermosensing role. Under oxidative stress conditions, yeast Mge1 is reversibly inactivated by methionine oxidation and reactivated by the MsrA/MsrB methionine sulfoxide reductases. This Mge1-Msr circuit modulates nucleotide exchange and protein import efficiency in response to matrix ROS, providing a physiological precedent for redox-tuned NEF activity that complements the vertebrate GrpEL2 observations (Allu et al., 2015; Needs et al., 2021; Poveda-Huertes et al., 2021).

## Potential GrpEL1-GrpEL2 heterodimer

Given their high sequence identity, structural conservation, and co-localization within the MM, it is not unreasonable to speculate that GrpEL1 and GrpEL2 may form hetero-oligomeric complexes under certain conditions. Indeed, there is evidence supporting the formation of a human GrpEL1-GrpEL2 hetero-oligomer. One such study identified a GrpEL1-GrpEL2 hetero-oligomer that exhibited enhanced thermostability compared to either GrpEL1 or GrpEL2 homodimers (Srivastava et al., 2017). The authors propose that the formation of the hetero-oligomeric complex may increase the solubility of the aggregation-prone GrpEL1/2 proteins. They further demonstrate that this hetero-oligomer can associate with the Tim44 scaffold at the IMM and that this species retains its co-chaperoning activity of mtHsp70. This species has been proposed to be preferentially recruited to the PAM complex in environments of oxidative stress.

However, in an analysis involving several human cell lines, the presence of a human GrpEL1-GrpEL2 hetero-oligomer could not be detected, and only homodimers of GrpEL1 and GrpEL2 could be identified (Konovalova et al., 2018). Given the structural plasticity of GrpEL1 and its ability to form homodimers or hetero-oligomers with GrpEL2, it is possible that different GrpEL1-containing complexes are recruited under basal versus stress conditions. Taken together, the possibility of GrpEL1-GrpEL2 hetero-oligomerization adds another layer of regulatory complexity to this system, reinforcing the broader theme of GrpE-like NEFs as stress-responsive modulators of proteostasis. However, further studies investigating the significance, function, and abundance of a GrpEL1-GrpEL2 hetero-oligomer in the context of mtHsp70 would provide valuable insights into the role of hetero-oligomerization in mitochondrial import and the maintenance of mitochondrial homeostasis.

## Regulation of GrpEL1 and GrpEL2 expression and stability

Beyond their structural and mechanistic distinctions, GrpEL1 and GrpEL2 are also subject to differential regulation at the transcriptional, translational, and possibly post-translational levels. Early transcriptomic analyses have indicated that GrpEL1 is broadly expressed across tissues, with highest abundance in metabolically active organs such as the heart, brain, kidney, and liver (Konovalova et al., 2018). In contrast, GrpEL2 exhibits a more restricted and variable expression profile, with relative enrichment in the pancreas, spleen, cerebrum, and thymus, suggesting a potential role in immune function or specialized metabolic pathways (Konovalova et al., 2018). To date, the regulatory mechanisms governing GrpEL1 and GrpEL2 expression remain largely unexplored. It is unknown whether either gene is induced in response to mitochondrial unfolded protein stress (UPR^mt^), oxidative stress, or other cellular insults that perturb proteostasis. Promoter analyses and epigenomic profiling may reveal the presence of stress-responsive transcription factor binding motifs, such as those for CHOP, ATF4, or NRF2, which are commonly activated in response to mitochondrial dysfunction. Single-cell and tissue datasets suggest these patterns are conserved across model organisms (e.g., mouse, zebrafish) with cell-type-specific tuning in high-metabolic or immune contexts (Jain et al., 2025; Ma et al., 2022; Needs et al., 2021; Schmidt et al., 2010).

At the protein level, both GrpEL1 and GrpEL2 are aggregation-prone *in vitro*, and their solubility is enhanced by co-expression or hetero-oligomerization. This suggests that expression balance and folding quality control are important determinants of GrpEL1/2 function. It is possible that mitochondrial proteases such as Lon or ClpXP modulate their turnover under stress conditions, though this remains speculative. Additionally, GrpEL2’s redox-sensitive Cys87 may also serve a regulatory role beyond functional modulation, potentially influencing its stability or oligomeric state in oxidative environments. Whether oxidative modification of GrpEL2 triggers ­degradation, sequestration, or altered import remains to be tested. These regulatory dimensions underscore the complexity of maintaining NEF homeostasis in mitochondria and highlight the need for system-level analyses of GrpEL1/2 abundance, turnover, and stress responsiveness.

## Disease associations and biomedical implications

Although the roles of GrpEL1 and GrpEL2 have primarily been studied in the context of fundamental mitochondrial biology, emerging evidence points to their potential involvement in human disease. Loss-of-function studies in animal models underscore the essentiality of GrpEL1, and these phenotypes are consistent with its central role in matrix protein import and maintenance of mitochondrial proteostasis (Ma et al., 2022; Manjunath et al., 2024; Neupane et al., 2022; Srivastava et al., 2017). Given the high-metabolic demands of muscle and neuronal tissues, impaired GrpEL1 function may contribute to or exacerbate pathologies in diseases such as mitochondrial myopathies and neurodegenerative disorders, with phenotypes likely reflecting combined defects in preprotein translocation, folding in the MM, and the maturation/assembly of oxidative phosphorylation components. GrpEL1 has also been implicated in human genetic diseases (Chen et al., 2019; Xie et al., 2025), and GrpEL1 expression may be altered in cancer cells that exhibit reprogrammed mitochondrial metabolism (Chen et al., 2019). Whether GrpEL1 is dysregulated to support increased protein import or modulated to resist apoptosis remains to be determined.

GrpEL2, although dispensable in cell lines, may play a more prominent role in stress adaptation. The discovery that its redox-sensitive cysteine can modulate binding to mtHsp70 raises the possibility that GrpEL2 acts as a regulatory buffer under oxidative conditions (Konovalova et al., 2018; Manjunath et al., 2024; Srivastava et al., 2017). In long-lived species or tissues that may be exposed to high amounts of ROS, such as immune cells or neurons, GrpEL2 might serve as a fine-tuner of mitochondrial proteostasis. While direct links to disease are currently lacking, perturbation of this redox regulation could theoretically influence susceptibility to conditions involving mitochondrial oxidative stress, such as Parkinson’s disease, Alzheimer’s disease, or certain cancers.

In the cancer context, emerging evidence suggests that mitochondrial chaperones, including mtHsp70, may be co-opted to support tumorigenesis (Albakova et al., 2020; Shen et al., 2025). It is conceivable that altered expression of GrpEL1 or GrpEL2 contributes to this reprogramming. Targeting their interaction surfaces or redox-switch mechanisms could thus represent a novel therapeutic strategy for modulating mitochondrial protein homeostasis in disease.

## Outstanding questions and future directions

Over the past two decades, significant progress has been made in elucidating the structural mechanisms underlying the function of GrpE-like NEFs. However, several important questions remain unresolved. First, while the structural basis for nucleotide exchange and substrate release has been clarified, the precise mechanism by which ATP binding drives complex dissociation from Hsp70 and triggers substrate release is incompletely defined. Addressing this will require kinetic studies that capture the full chaperone cycle using labeled substrates and real-time tracking. Second, the role of GrpEL1 in the PAM complex remains incompletely mapped. Although interaction with Tim44 has been suggested, the full set of physical and functional interactions between GrpEL1 and other PAM components—including Pam18, Pam16, and mtHsp70—has not been resolved (Srivastava et al., 2017). Determining how GrpEL1 is spatially recruited and retained at the inner membrane could yield critical insights into the regulation of matrix import. Third, the physiological relevance of GrpEL2 remains an open question. While GrpEL1 is essential, GrpEL2’s role appears more nuanced. Is it required only under stress conditions? Does it chaperone distinct substrates or contribute to specific mitochondrial functions? Likewise, the proposed GrpEL1-GrpEL2 hetero-oligomer remains controversial, with conflicting data regarding its presence and function in cells. Finally, the broader role of NEFs as regulatory hubs, rather than passive exchange factors, is gaining traction. Redox regulation, conformational tuning, and stress-responsive modulation all point to a more active role in controlling chaperone cycles. Future investigations using time-resolved cryoEM, *in situ* crosslinking mass spectrometry, and proximity-based interactome mapping will be essential for disentangling these dynamic regulatory mechanisms.

As GrpE-like proteins continue to reveal layers of complexity, they serve as a paradigm for understanding how core molecular machines evolve, diversify, and adapt to maintain homeostasis in increasingly complex cellular environments.

## References

[pwaf086-B1] Abramson J , Adler J, Dunger J et al Accurate structure prediction of biomolecular interactions with AlphaFold 3. Nature 2024;630:493–500. 38718835 10.1038/s41586-024-07487-w PMC11168924

[pwaf086-B2] Albakova Z , Armeev GA, Kanevskiy LM et al HSP70 multi-functionality in cancer. Cells 2020;9:587. 32121660 10.3390/cells9030587 PMC7140411

[pwaf086-B3] Alberti S , Esser C, Höhfeld J. BAG-1—a nucleotide exchange factor of Hsc70 with multiple cellular functions. Cell Stress Chaper 2003;8:225. 10.1379/1466-1268(2003)008<0225:bnefoh>2.0.co;2 PMC514875 14984055

[pwaf086-B4] Allu PK , Marada A, Boggula Y et al Methionine sulfoxide reductase 2 reversibly regulates Mge1, a cochaperone of mitochondrial Hsp70, during oxidative stress. Mol Biol Cell 2015;26:406–419. 25428986 10.1091/mbc.E14-09-1371 PMC4310733

[pwaf086-B5] Bracher A , Verghese J. Nucleotide exchange factors for Hsp70 molecular chaperones: GrpE, Hsp110/Grp170, HspBP1/Sil1, and BAG Domain Proteins. In: Edkins AL, Blatch GL (eds.), The Networking of Chaperones by Co-Chaperones, Vol. 101. Cham: Springer International Publishing, 2023, 1–39. 10.1007/978-3-031-14740-1_1 36520302

[pwaf086-B6] Brehmer D , Gässler C, Rist W et al Influence of GrpE on DnaK-substrate interactions. J Biol Chem 2004;279:27957–27964. 15102842 10.1074/jbc.M403558200

[pwaf086-B7] Calloni G , Chen T, Schermann SM et al DnaK functions as a Central hub in the *E. coli* chaperone network. Cell Rep 2012;1:251–264. 22832197 10.1016/j.celrep.2011.12.007

[pwaf086-B8] Chen P , Wang B, Mo Q et al The LIV-1-GRPEL1 axis adjusts cell fate during anti-mitotic agent-damaged mitosis. EBioMedicine 2019;49:26–39. 31636012 10.1016/j.ebiom.2019.09.054 PMC6945280

[pwaf086-B9] Clerico EM , Tilitsky JM, Meng W et al How Hsp70 molecular machines interact with their substrates to mediate diverse physiological functions. J Mol Biol 2015;427:1575–1588. 25683596 10.1016/j.jmb.2015.02.004 PMC4440321

[pwaf086-B10] Craig EA. Hsp70 at the membrane: driving protein translocation. BMC Biol 2018;16:11. 29343244 10.1186/s12915-017-0474-3 PMC5773037

[pwaf086-B11] Den Brave F , Schulte U, Fakler B et al Mitochondrial complexome and import network. Trends Cell Biol 2024;34:578–594. 37914576 10.1016/j.tcb.2023.10.004

[pwaf086-B12] Dragovic Z , Broadley SA, Shomura Y et al Molecular chaperones of the Hsp110 family act as nucleotide exchange factors of Hsp70s. EMBO J 2006;25:2519–2528. 16688212 10.1038/sj.emboj.7601138 PMC1478182

[pwaf086-B13] Fernández-Fernández MR , Gragera M, Ochoa-Ibarrola L et al Hsp70—a master regulator in protein degradation. FEBS Lett 2017;591:2648–2660. 28696498 10.1002/1873-3468.12751

[pwaf086-B14] Gelinas AD , Langsetmo K, Toth J et al A structure-based interpretation of *E. coli* GrpE thermodynamic properties. J Mol Biol 2002;323:131–142. 12368105 10.1016/s0022-2836(02)00915-4

[pwaf086-B15] Gill-Hille M , Wang A, Murcha MW. Presequence translocase-associated motor subunits of the mitochondrial protein import apparatus are dual-targeted to mitochondria and plastids. Front Plant Sci 2022;13:981552. 36438081 10.3389/fpls.2022.981552 PMC9695410

[pwaf086-B16] Grimshaw JPA , Jelesarov I, Schönfeld H-J et al Reversible thermal transition in GrpE, the nucleotide exchange factor of the DnaK Heat-Shock system. J Biol Chem 2001;276:6098–6104. 11084044 10.1074/jbc.M009290200

[pwaf086-B17] Groemping Y , Reinstein J. Folding properties of the nucleotide exchange factor GrpE from *Thermus thermophilus*: GrpE is a thermosensor that mediates heat shock response. J Mol Biol 2001;314:167–178. 11724541 10.1006/jmbi.2001.5116

[pwaf086-B18] Harrison C. GrpE, a nucleotide exchange factor for DnaK. Cell Stress Chaper 2003;8:218. 10.1379/1466-1268(2003)008<0218:ganeff>2.0.co;2 PMC514874 14984054

[pwaf086-B19] Harrison CJ , Hayer-Hartl M, Liberto MD et al Crystal structure of the nucleotide exchange factor GrpE bound to the ATPase domain of the molecular chaperone DnaK. Science 1997;276:431–435. 9103205 10.1126/science.276.5311.431

[pwaf086-B20] Hewitt V , Alcock F, Lithgow T. Minor modifications and major adaptations: the evolution of molecular machines driving mitochondrial protein import. Biochim Biophys Acta 2011;1808:947–954. 20659421 10.1016/j.bbamem.2010.07.019

[pwaf086-B21] Hewitt V , Lithgow T, Waller RF. Modifications and innovations in the evolution of mitochondrial protein import pathways. In Löffelhardt W (ed.), Endosymbiosis. Vienna: Springer, 2014, 19–35.

[pwaf086-B22] Hutu DP , Guiard B, Chacinska A et al Mitochondrial protein import motor: differential role of Tim44 in the recruitment of Pam17 and J-Complex to the presequence translocase. Mol Biol Cell 2008;19:2642–2649. 18400944 10.1091/mbc.E07-12-1226 PMC2397324

[pwaf086-B23] Jain N , Chacinska A, Rehling P. Understanding mitochondrial protein import: a revised model of the presequence translocase. Trends Biochem Sci 2025;50:585–595. 40155273 10.1016/j.tibs.2025.03.001

[pwaf086-B24] Kampinga HH , Craig EA. The HSP70 chaperone machinery: J proteins as drivers of functional specificity. Nat Rev Mol Cell Biol 2010;11:579–592. 20651708 10.1038/nrm2941 PMC3003299

[pwaf086-B25] Kityk R , Kopp J, Mayer MP. Molecular mechanism of J-domain-triggered ATP hydrolysis by Hsp70 chaperones. Mol Cell 2018;69:227–237.e4. 29290615 10.1016/j.molcel.2017.12.003

[pwaf086-B26] Konovalova S , Liu X, Manjunath P et al Redox regulation of GRPEL2 nucleotide exchange factor for mitochondrial HSP70 chaperone. Redox Biol 2018;19:37–45. 30098457 10.1016/j.redox.2018.07.024 PMC6089081

[pwaf086-B27] Li Y , Dudek J, Guiard B et al The presequence translocase-associated protein import motor of mitochondria. J Biol Chem 2004;279:38047–38054. 15218029 10.1074/jbc.M404319200

[pwaf086-B28] Ma C , Gao B, Wang Z et al GrpEL1 regulates mitochondrial unfolded protein response after experimental subarachnoid hemorrhage *in vivo* and *in vitro*. Brain Res Bull 2022;181:97–108. 35093469 10.1016/j.brainresbull.2022.01.014

[pwaf086-B29] Macario AJL , Brocchieri L, Shenoy AR et al Evolution of a protein-folding machine: genomic and evolutionary analyses reveal three lineages of the archaeal hsp70 (dnaK) gene. J Mol Evol 2006;63:74–86. 16788741 10.1007/s00239-005-6207-1

[pwaf086-B30] Manjunath P , Stojkovič G, Euro L et al Preferential binding of ADP-bound mitochondrial HSP70 to the nucleotide exchange factor GRPEL1 over GRPEL2. Protein Sci 2024;33:e5190. 39445986 10.1002/pro.5190 PMC11500471

[pwaf086-B31] Mayer MP , Bukau B. Hsp70 chaperones: cellular functions and molecular mechanism. Cell Mol Life Sci 2005;62:670–684. 15770419 10.1007/s00018-004-4464-6 PMC2773841

[pwaf086-B32] Mayer MP , Gierasch LM. Recent advances in the structural and mechanistic aspects of Hsp70 molecular chaperones. J Biol Chem 2019;294:2085–2097. 30455352 10.1074/jbc.REV118.002810 PMC6369304

[pwaf086-B33] Michaelis JB , Brunstein ME, Bozkurt S et al Protein import motor complex reacts to mitochondrial misfolding by reducing protein import and activating mitophagy. Nat Commun 2022;13:5164. 36056001 10.1038/s41467-022-32564-x PMC9440083

[pwaf086-B34] Morizono MA , McGuire KL, Birouty NI et al Structural insights into GrpEL1-mediated nucleotide and substrate release of human mitochondrial Hsp70. Nat Commun 2024;15:10815. 39737924 10.1038/s41467-024-54499-1 PMC11685456

[pwaf086-B35] Moro F , Taneva SG, Velázquez-Campoy A et al GrpE N-terminal domain contributes to the interaction with DnaK and modulates the dynamics of the chaperone substrate binding domain. J Mol Biol 2007;374:1054–1064. 17976642 10.1016/j.jmb.2007.10.002

[pwaf086-B36] Nakamura A , Takumi K, Miki K. Crystal structure of a thermophilic GrpE protein: insight into thermosensing function for the DnaK chaperone system. J Mol Biol 2010;396:1000–1011. 20036249 10.1016/j.jmb.2009.12.028

[pwaf086-B37] Needs HI , Protasoni M, Henley JM et al Interplay between mitochondrial protein import and respiratory complexes assembly in neuronal health and degeneration. Life 2021;11:432. 34064758 10.3390/life11050432 PMC8151517

[pwaf086-B38] Neupane N , Rajendran J, Kvist J et al Inter-organellar and systemic responses to impaired mitochondrial matrix protein import in skeletal muscle. Commun Biol 2022;5:1060. 36198903 10.1038/s42003-022-04034-z PMC9534917

[pwaf086-B39] Poveda-Huertes D , Taskin AA, Dhaouadi I et al Increased mitochondrial protein import and cardiolipin remodelling upon early mtUPR. PLoS Genet 2021;17:e1009664. 34214073 10.1371/journal.pgen.1009664 PMC8282050

[pwaf086-B40] Priesnitz C , Böttinger L, Zufall N et al Coupling to Pam16 differentially controls the dual role of Pam18 in protein import and respiratory chain formation. Cell Rep 2022;39:110619. 35385740 10.1016/j.celrep.2022.110619

[pwaf086-B41] Roger AJ , Muñoz-Gómez SA, Kamikawa R. The origin and diversification of mitochondria. Curr Biol 2017;27:R1177–R1192. 29112874 10.1016/j.cub.2017.09.015

[pwaf086-B42] Rosenzweig R , Nillegoda NB, Mayer MP et al The Hsp70 chaperone network. Nat Rev Mol Cell Biol 2019;20:665–680. 31253954 10.1038/s41580-019-0133-3

[pwaf086-B43] Schmidt O , Pfanner N, Meisinger C. Mitochondrial protein import: from proteomics to functional mechanisms. Nat Rev Mol Cell Biol 2010;11:655–667. 20729931 10.1038/nrm2959

[pwaf086-B44] Schönfeld H-J , Schmidt D, Schröder H et al The DnaK chaperone system of *Escherichia coli*: quaternary structures and interactions of the DnaK and GrpE components. J Biol Chem 1995;270:2183–2189. 7836448 10.1074/jbc.270.5.2183

[pwaf086-B45] Shen N , Xia Y, Shen X et al HSPA9 contributes to tumor progression and ferroptosis resistance by enhancing USP14-driven SLC7A11 deubiquitination in multiple myeloma. Cell Rep 2025;44:115720. 40372919 10.1016/j.celrep.2025.115720

[pwaf086-B46] Shomura Y , Dragovic Z, Chang H-C et al Regulation of Hsp70 function by HspBP1. Mol Cell 2005;17:367–379. 15694338 10.1016/j.molcel.2004.12.023

[pwaf086-B47] Srivastava S , Savanur MA, Sinha D et al Regulation of mitochondrial protein import by the nucleotide exchange factors GrpEL1 and GrpEL2 in human cells. J Biol Chem 2017;292:18075–18090. 28848044 10.1074/jbc.M117.788463 PMC5672033

[pwaf086-B48] Szabo A , Langer T, Schröder H et al The ATP hydrolysis-dependent reaction cycle of the *Escherichia coli* Hsp70 system DnaK, DnaJ, and GrpE. Proc Natl Acad Sci U S A 1994;91:10345–10349. 7937953 10.1073/pnas.91.22.10345 PMC45016

[pwaf086-B49] Truscott KN , Voos W, Frazier AE et al A J-protein is an essential subunit of the presequence translocase–associated protein import motor of mitochondria. J Cell Biol 2003;163:707–713. 14638855 10.1083/jcb.200308004 PMC2173675

[pwaf086-B50] Van Der Laan M , Chacinska A, Lind M et al Pam17 is required for architecture and translocation activity of the mitochondrial protein import motor. Mol Cell Biol 2005;25:7449–7458. 16107694 10.1128/MCB.25.17.7449-7458.2005 PMC1190294

[pwaf086-B51] Voos W , Gambill BD, Laloraya S et al Mitochondrial GrpE is present in a complex with hsp70 and preproteins in transit across membranes. Mol Cell Biol 1994;14:6627–6634. 7935381 10.1128/mcb.14.10.6627-6634.1994 PMC359192

[pwaf086-B52] Wu C-C , Naveen V, Chien C-H et al Crystal structure of DnaK protein complexed with nucleotide exchange factor GrpE in DnaK chaperone system. J Biol Chem 2012;287:21461–21470. 22544739 10.1074/jbc.M112.344358 PMC3375567

[pwaf086-B53] Xiao X , Fay A, Molina PS et al Structure of the *M. tuberculosis* DnaK–GrpE complex reveals how key DnaK roles are controlled. Nat Commun 2024;15:660. 38253530 10.1038/s41467-024-44933-9 PMC10803776

[pwaf086-B54] Xie M , Wu X, Liu X et al GrpEL1 overexpression mitigates hippocampal neuron damage via mitochondrial unfolded protein response after experimental status epilepticus. Neurobiol Dis 2025;206:106838. 39938576 10.1016/j.nbd.2025.106838

[pwaf086-B55] Zylicz M , Ang D, Georgopoulos C. The grpE protein of *Escherichia coli*. Purification and properties. J Biol Chem 1987;262:17437–17442. 2826421

[pwaf086-B56] Zylicz M , Ang D, Liberek K et al Initiation of lambda DNA replication with purified host- and bacteriophage-encoded proteins: the role of the dnaK, dnaJ and grpE heat shock proteins. EMBO J 1989;8:1601–1608. 2527744 10.1002/j.1460-2075.1989.tb03544.x PMC400992

